# Crystal structure of di­aqua­tris­(1-ethyl-1*H*-imidazole-κ*N* ^3^)(sulfato-κ*O*)nickel(II)

**DOI:** 10.1107/S2056989016002863

**Published:** 2016-02-20

**Authors:** Tamas Holczbauer, Attila Domjan, Csaba Fodor

**Affiliations:** a Chemical Crystallography Research Group, Institute of Organic Chemistry, Hungarian Academy of Sciences, Magyar Tudosok Korutja 2, Budapest H-1117, Hungary; b NMR Research Group, Institute of Organic Chemistry, Hungarian Academy of Sciences, Magyar Tudosok Korutja 2, Budapest H-1117, Hungary; c Polymer Chemistry Research Group, Institute of Materials and Environmental Chemistry, Research Centre for Natural Sciences, Hungarian Academy of Sciences, Magyar Tudosok Korutja 2, Budapest H-1117, Hungary

**Keywords:** crystal structure, nickel(II) complex, hydrogen bonding, disorder, coordination

## Abstract

In the title complex, [Ni(SO_4_)(C_5_H_8_N_2_)_3_(H_2_O)_2_], the Ni^II^ ion is coordinated by three facial 1-ethyl-1*H*-imidazole ligands, one monodentate sulfate ligand and two water mol­ecules in a slightly distorted octa­hedral coordination environment. In the crystal, two pairs of O—H⋯O hydrogen bonds link complex mol­ecules, forming inversion dimers incorporating *R*_2_^4^(8), *R*_2_^2^(8) and *R*_2_^2^(12) rings. The dimeric unit also contains two symmetry-unique intra­molecular O—H⋯O hydrogen bonds. In addition, weak C—H⋯O hydrogen bonds, weak C—H⋯π inter­actions and π–π inter­actions with a centroid–centroid distance of 3.560 (2) Å combine to form a three-dimensional network. One of the ethyl groups is disordered over two sets of sites with occupancies in the ratio 0.586 (7):0.414 (7).

## Abstract 2

The Ni^II^ ion atom is octa­hedrally coordinated in di­aqua­tris­(1-ethyl-1*H*-imidazole)­sulfato­nickel(II). There are three organic ligands, two water and the sulfate anion coordinated around the Ni^II^ centre. Two complex mol­ecules form an inversion dimer *via* two pairs of O—H⋯O hydrogen bonds between the coordinating sulfate anion and a water mol­ecule in the unit cell.

## Chemical context   

In spite of efforts in the past decades to synthesize structurally highly varying metal-organic complexes, no structures up to this point have been reported which contain the combination of a hydro­philic sulfate anion, water mol­ecules and hydro­phobic 1-ethyl-1*H*-imidazole mol­ecules as ligands. The title compound was prepared by the reaction of NiSO_4_·6H_2_O and 1-ethyl-1*H*-imidazole. The crystal structure of the title compound is presented herein.
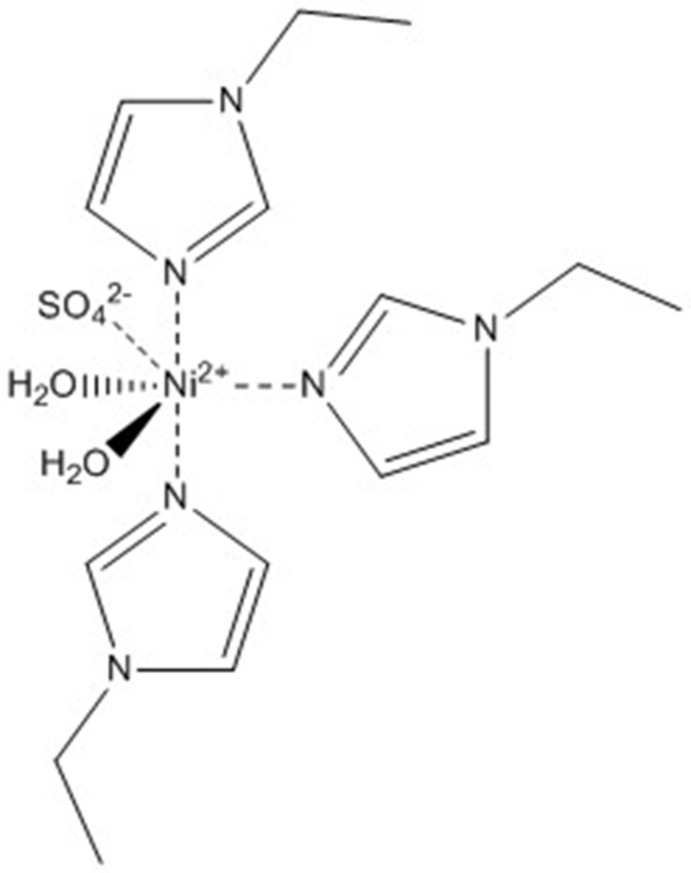


## Structural commentary   

**Figure 1:**
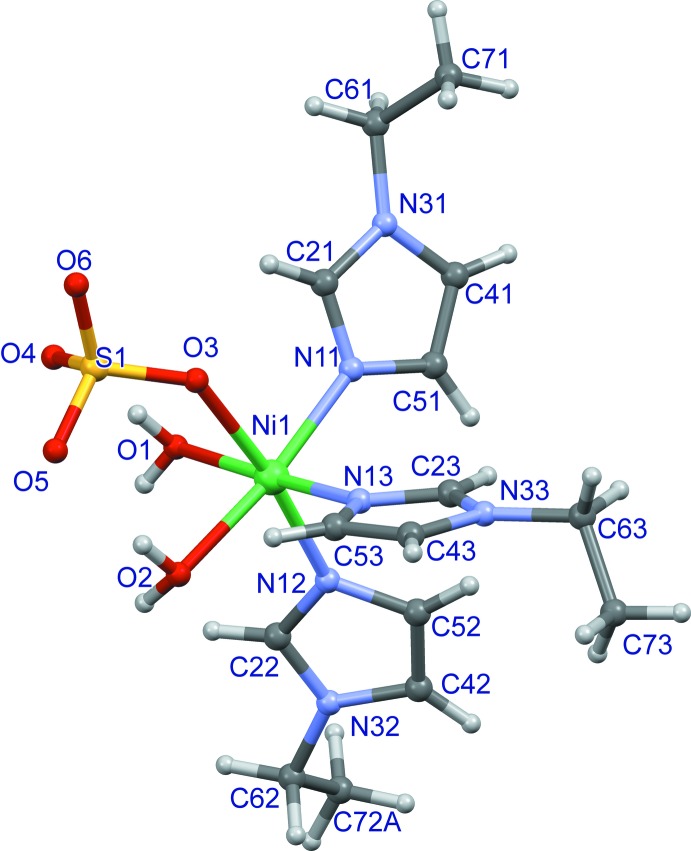
The mol­ecular structure of the title compound, with displacement ellipsoids drawn at the 50% probability level. Only the major component of disorder is shown.

**Figure 2:**
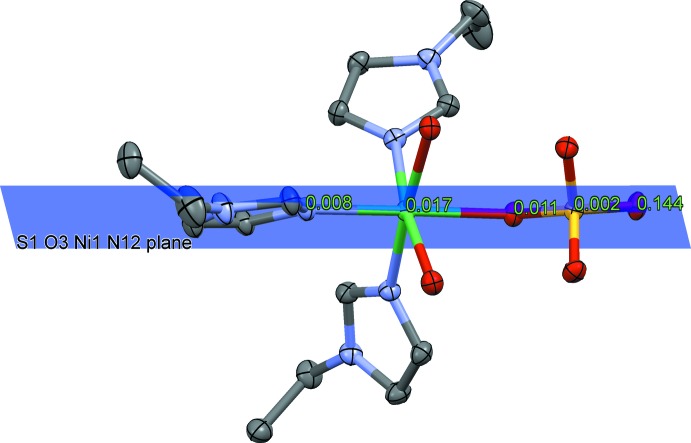
The distances of the atoms N12, Ni1, O3, S1 and O6 from the least-squares plane defined by S1/O3/Ni1/N12.

The mol­ecular structure of the title compound is shown in Fig. 1 ▸. The Ni^II^ ion is coordinated in a slightly distorted octa­hedral geometry by three facially arranged 1-ethyl-1*H*-imidazole ligands, one monodentate sulfate ligand and two water mol­ecules. The Ni—N bond lengths are in the range 2.0630 (16)–2.0817 (17)Å and the Ni—O bond lengths are in the range 2.1195 (15)–2.1502 (14). The Ni^ii^ ion is displaced by 0.1038 (3) Å from the O1/O2/N11/N13 plane. The distances of two water O atoms O1 and O2 from the S1/O3/Ni1/N12 plane are the same within experimental error, with values of 1.520 (2) and −1.504 (2) Å, respectively. The sulfate atom O6 is displaced by only 0.144 (2) Å from the S1/O3/Ni1/N12 plane, while atoms O4 and O5 are displaced by 1.114 (2) and −1.298 (2) Å, respectively, from this plane (see Fig. 2 ▸.).

## Supra­molecular features   

**Figure 3:**
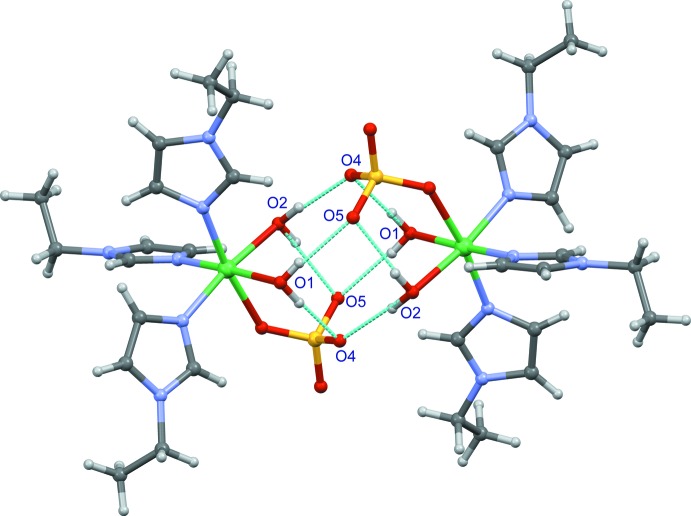
An inversion dimer of the title compound. Hydrogen bonds are shown as dotted blue lines.

In the crystal, two pairs of O—H⋯O hydrogen bonds (Table 1 ▸) link complex mol­ecules, forming inversion dimers incorporating 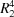
(8), 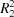
(8) and 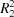
(12) rings. The dimeric unit also contains two symmetry-unique intra­molecular O—H⋯O hydrogen bonds (Fig. 3 ▸). In addition, weak C—H⋯O hydrogen bonds, weak C—H⋯π inter­actions and π–π inter­actions with a centroid–centroid distance of 3.560 (2) Å combine to form a three-dimensional network. The π–π inter­action is observed between the N11/C21/N31/C41/C51 ring and the inversion-related ring at (1 − *x*, −*y*, 1 − *z*).

## Database survey   

A search of the Cambridge Structural Database (CSD; Groom & Allen, 2014 ▸) for mol­ecules with two water ligands, a sulfate anion and three nitro­gen-containing mol­ecules gave the following hits with Ni: ARUZIW (Ouyang *et al.*, 2004 ▸), BEDSEJ (Wan *et al.*, 2003 ▸), FOXRAM (Xu *et al.*, 2009 ▸), REHKUL (Díaz de Vivar *et al.*, 2006 ▸), ZAMFUO (Mukherjee *et al.*, 1995 ▸), and with Cu: ODAHEI, ODAHOS (Adarsh *et al.*, 2011 ▸), XIHSAI (Gómez-Saiz *et al.*, 2002 ▸) and QUSJAP (Calatayud *et al.*, 2000 ▸).

A similar type of hydrogen bonding occurs between the sulfate anion and water mol­ecules in the complex BEDSEJ. In ARUZIW, one of the hydrogen bonds of the sulfate anion involves the protonated hydrogen-acceptor nitro­gen atom. Unlike the title compound, one of the water ligands in FOXRAM, REHKUL and ZAMFUO is *trans* to the sulfate ligand. This also the case in the copper-containing structure QUSJUP, but in ODAHEI, ODAHOS and XIHSAI the two aqua ligands are *trans* to each other.

Complexes with one Ni^II^ ion and at least three 1-ethyl-1*H*-imidazole ligands have already been reported in the literature (DEDLIJ: Huxel *et al.*, 2012 ▸; IDEJAE: Çetinkaya *et al.*, 2013 ▸; WENYAK: Liu *et al.*, 2006 ▸). Complexes have also been reported for Cu (GEVGEV: Hoogerstraete *et al.*, 2012 ▸; UFOMIM: Liu *et al.*, 2008 ▸; XIKXEV: Liu *et al.*, 2007 ▸).

## Synthesis and crystallization   

NiSO_4_·6H_2_O and 1-ethyl-1*H*-imidazole in a 1:1 stoichiometric ratio formed an exothermic reaction. The compound was dissolved in methanol and the solution was precipitated with ethyl acetate. After one week, blue crystals suitable for X-ray diffraction grew in the vessel.

## Refinement   

Crystal data, data collection and structure refinement details are summarized in Table 2 ▸. Six reflections were found to be shaded by the beamstop and removed from the data set. The hydrogen atoms of the water mol­ecules were located in a difference map and refined freely. Hydrogen atoms bonded to C atoms were placed in calculated positions and refined in a riding-model approximation. One of the ethyl groups is disordered over two sets of sites with occupancies in the ratio 0.586 (7):0.414 (7).

## Supplementary Material

Crystal structure: contains datablock(s) I. DOI: 10.1107/S2056989016002863/lh5802sup1.cif

Structure factors: contains datablock(s) I. DOI: 10.1107/S2056989016002863/lh5802Isup2.hkl

CCDC reference: 1454040

Additional supporting information:  crystallographic information; 3D view; checkCIF report

## Figures and Tables

**Table 1:** Hydrogen-bond geometry (Å, °) *Cg*1 is the centroid of the N13–C23–N33–C43–C53 ring and *Cg*2 is the centroid of the N12–C22–N32–C42–C52 ring

*D*—H⋯*A*	*D*—H	H⋯*A*	*D*⋯*A*	*D*—H⋯*A*
O1—H1*A*⋯O4	0.84 (3)	1.88 (3)	2.706 (2)	170 (3)
O1—H1*B*⋯O5^i^	0.77 (3)	2.02 (3)	2.786 (2)	173 (3)
O2—H2*B*⋯O4^i^	0.85 (3)	1.88 (3)	2.720 (2)	171 (3)
O2—H2*A*⋯O5	0.81 (3)	2.00 (3)	2.791 (2)	165 (3)
C22—H22⋯O5^i^	0.95	2.60	3.511 (3)	162
C23—H23⋯O6^ii^	0.95	2.56	3.409 (3)	150
C52—H52⋯O6^ii^	0.95	2.42	3.315 (3)	157
C73—H73*B*⋯O6^iii^	0.98	2.40	3.347 (3)	163
C61—H61*A*⋯*Cg*1^iv^	0.99	2.80	3.779 (3)	169
C61—H61*B*⋯*Cg*2^v^	0.99	2.97	3.816 (3)	144

Symmetry codes: (i) 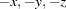; (ii) 
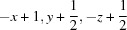
; (iii) 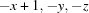; (iv) 
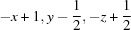
; (v) 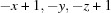.

**Table 2:** Experimental details

Crystal data	
Chemical formula	[Ni(SO_4_)(C_5_H_8_N_2_)_3_(H_2_O)_2_]
*M*_r_	478.97
Crystal system, space group	Monoclinic, *P*2_1_/*c*
Temperature (K)	131
*a*, *b*, *c* (Å)	12.0252 (13), 14.3481 (15), 15.3502 (11)
β (°)	128.980 (5)
*V* (Å^3^)	2058.9 (4)
*Z*	4
Radiation type	Mo *K*α
μ (mm^−1^)	1.09
Crystal size (mm)	0.40 × 0.25 × 0.15

Data collection	
Diffractometer	Rigaku R-AXIS RAPID-S
Absorption correction	Numerical (*NUMABS*; Higashi, 1999 ▸)
*T*_min_, *T*_max_	0.705, 1.000
No. of measured, independent and observed [*I* > 2σ(*I*)] reflections	28931, 4723, 4284
*R*_int_	0.030
(sin θ/λ)_max_ (Å^−1^)	0.649

Refinement	
*R*[*F* ^2^ > 2σ(*F* ^2^)], *wR*(*F* ^2^), *S*	0.033, 0.085, 1.07
No. of reflections	4723
No. of parameters	290
No. of restraints	2
H-atom treatment	H atoms treated by a mixture of independent and constrained refinement
Δρ_max_, Δρ_min_ (e Å^−3^)	1.04, −1.12

Computer programs: *CrystalClear* (Rigaku, 2008 ▸), *SHELXS97* (Sheldrick, 2008 ▸), *SHELXL2014* (Sheldrick, 2015 ▸), *Mercury* (Macrae *et al.*, 2008 ▸) and *PLATON* (Spek, 2009 ▸).

## supplementary crystallographic information

### Crystal data

**Table:**

[Ni(SO_4_)(C_5_H_8_N_2_)_3_(H_2_O)_2_]	*F*(000) = 1008
*M**_r_* = 478.97	*D*_x_ = 1.545 Mg m^−^^3^
Monoclinic, *P*2_1_/*c*	Mo *K*α radiation, λ = 0.71075 Å
*a* = 12.0252 (13) Å	Cell parameters from 24228 reflections
*b* = 14.3481 (15) Å	θ = 3.0–29.2°
*c* = 15.3502 (11) Å	µ = 1.09 mm^−^^1^
β = 128.980 (5)°	*T* = 131 K
*V* = 2058.9 (4) Å^3^	Prism, blue–green
*Z* = 4	0.40 × 0.25 × 0.15 mm

### Data collection

**Table:**

Rigaku R-AXIS RAPID-S diffractometer	4723 independent reflections
Radiation source: NORMAL-focus sealed tube	4284 reflections with *I* > 2σ(*I*)
Graphite monochromator	*R*_int_ = 0.030
Detector resolution: 10.0000 pixels mm^-1^	θ_max_ = 27.5°, θ_min_ = 3.0°
dtprofit.ref scans	*h* = −15→15
Absorption correction: numerical (*NUMABS*; Higashi, 1999)	*k* = −18→18
*T*_min_ = 0.705, *T*_max_ = 1.000	*l* = −19→19
28931 measured reflections	

### Refinement

**Table:**

Refinement on *F*^2^	Primary atom site location: structure-invariant direct methods
Least-squares matrix: full	Secondary atom site location: structure-invariant direct methods
*R*[*F*^2^ > 2σ(*F*^2^)] = 0.033	Hydrogen site location: mixed
*wR*(*F*^2^) = 0.085	H atoms treated by a mixture of independent and constrained refinement
*S* = 1.07	*w* = 1/[σ^2^(*F*_o_^2^) + (0.0358*P*)^2^ + 2.7059*P*] where *P* = (*F*_o_^2^ + 2*F*_c_^2^)/3
4723 reflections	(Δ/σ)_max_ = 0.001
290 parameters	Δρ_max_ = 1.04 e Å^−^^3^
2 restraints	Δρ_min_ = −1.12 e Å^−^^3^

### Special details

**Table:**

Geometry. All e.s.d.'s (except the e.s.d. in the dihedral angle between two l.s. planes) are estimated using the full covariance matrix. The cell e.s.d.'s are taken into account individually in the estimation of e.s.d.'s in distances, angles and torsion angles; correlations between e.s.d.'s in cell parameters are only used when they are defined by crystal symmetry. An approximate (isotropic) treatment of cell e.s.d.'s is used for estimating e.s.d.'s involving l.s. planes.

### Fractional atomic coordinates and isotropic or equivalent isotropic displacement parameters (Å^2^)

**Table:**

	*x*	*y*	*z*	*U*_iso_*/*U*_eq_	Occ. (<1)
Ni1	0.32786 (2)	0.06839 (2)	0.17394 (2)	0.01465 (8)	
S1	0.19425 (5)	−0.13289 (3)	0.04041 (4)	0.01533 (11)	
O1	0.17269 (16)	0.02378 (11)	0.19192 (12)	0.0184 (3)	
O2	0.16244 (15)	0.09393 (11)	0.00106 (12)	0.0194 (3)	
O3	0.31939 (14)	−0.07108 (9)	0.11877 (12)	0.0181 (3)	
O4	0.10655 (15)	−0.13787 (10)	0.07794 (12)	0.0203 (3)	
O5	0.10552 (15)	−0.09040 (10)	−0.07368 (12)	0.0205 (3)	
O6	0.24281 (15)	−0.22575 (10)	0.03973 (12)	0.0226 (3)	
N11	0.48566 (17)	0.02439 (12)	0.33618 (13)	0.0171 (3)	
N12	0.30580 (17)	0.20374 (12)	0.21002 (14)	0.0186 (3)	
N13	0.47676 (17)	0.10307 (12)	0.15321 (14)	0.0183 (3)	
N31	0.61057 (18)	−0.07752 (12)	0.47365 (15)	0.0205 (3)	
N32	0.1943 (2)	0.33190 (13)	0.19606 (18)	0.0293 (4)	
N33	0.67307 (18)	0.15163 (12)	0.18436 (15)	0.0203 (3)	
C21	0.5071 (2)	−0.06505 (14)	0.36263 (17)	0.0182 (4)	
H21	0.4561	−0.1143	0.3101	0.022*	
C22	0.1814 (2)	0.24081 (14)	0.17007 (19)	0.0235 (4)	
H22	0.0937	0.2074	0.1282	0.028*	
C23	0.6062 (2)	0.13902 (14)	0.22810 (17)	0.0200 (4)	
H23	0.6465	0.1540	0.3030	0.024*	
C41	0.6587 (2)	0.00910 (15)	0.52137 (17)	0.0215 (4)	
H41	0.7313	0.0229	0.5985	0.026*	
C42	0.3367 (2)	0.35474 (15)	0.2570 (2)	0.0280 (5)	
H42	0.3790	0.4141	0.2871	0.034*	
C43	0.5818 (2)	0.12208 (15)	0.07504 (18)	0.0239 (4)	
H43	0.5989	0.1225	0.0225	0.029*	
C51	0.5813 (2)	0.07121 (14)	0.43575 (17)	0.0211 (4)	
H51	0.5916	0.1370	0.4434	0.025*	
C52	0.4042 (2)	0.27554 (14)	0.26545 (18)	0.0215 (4)	
H52	0.5038	0.2701	0.3036	0.026*	
C53	0.4619 (2)	0.09216 (15)	0.05751 (17)	0.0209 (4)	
H53	0.3798	0.0673	−0.0110	0.025*	
C61	0.6605 (3)	−0.16783 (16)	0.5309 (2)	0.0306 (5)	
H61A	0.5983	−0.2177	0.4768	0.037*	
H61B	0.6524	−0.1688	0.5912	0.037*	
C62	0.0740 (3)	0.39212 (18)	0.1578 (3)	0.0462 (7)	
H62A	−0.0128	0.3533	0.1216	0.055*	0.586 (7)
H62B	0.0576	0.4354	0.1005	0.055*	0.586 (7)
H62C	0.0944	0.4235	0.2239	0.055*	0.414 (7)
H62D	−0.0116	0.3526	0.1243	0.055*	0.414 (7)
C63	0.8153 (2)	0.19343 (16)	0.24232 (19)	0.0254 (4)	
H63A	0.8754	0.1508	0.2368	0.030*	
H63B	0.8617	0.2014	0.3227	0.030*	
C71	0.8118 (3)	−0.18738 (19)	0.5804 (3)	0.0461 (7)	
H71A	0.8749	−0.1414	0.6389	0.069*	
H71B	0.8215	−0.1836	0.5217	0.069*	
H71C	0.8384	−0.2500	0.6131	0.069*	
C72A	0.0951 (4)	0.4480 (3)	0.2497 (3)	0.0344 (11)	0.586 (7)
H72A	0.0106	0.4869	0.2184	0.052*	0.586 (7)
H72B	0.1796	0.4878	0.2850	0.052*	0.586 (7)
H72C	0.1088	0.4057	0.3059	0.052*	0.586 (7)
C72B	0.0385 (8)	0.4659 (5)	0.0733 (6)	0.061 (3)	0.414 (7)
H72D	0.1231	0.5044	0.1042	0.092*	0.414 (7)
H72E	−0.0394	0.5052	0.0568	0.092*	0.414 (7)
H72F	0.0086	0.4358	0.0042	0.092*	0.414 (7)
C73	0.8063 (3)	0.28677 (17)	0.1928 (2)	0.0286 (5)	
H73A	0.7374	0.3265	0.1894	0.043*	
H73B	0.7752	0.2778	0.1171	0.043*	
H73C	0.9006	0.3166	0.2399	0.043*	
H2A	0.145 (3)	0.044 (2)	−0.030 (2)	0.030 (7)*	
H2B	0.083 (3)	0.112 (2)	−0.017 (2)	0.038 (8)*	
H1A	0.152 (3)	−0.029 (2)	0.163 (3)	0.041 (8)*	
H1B	0.098 (3)	0.046 (2)	0.159 (3)	0.035 (8)*	

### Atomic displacement parameters (Å^2^)

**Table:**

	*U*^11^	*U*^22^	*U*^33^	*U*^12^	*U*^13^	*U*^23^
Ni1	0.01215 (12)	0.01505 (13)	0.01544 (13)	−0.00015 (8)	0.00804 (10)	−0.00036 (9)
S1	0.0129 (2)	0.0156 (2)	0.0155 (2)	0.00119 (16)	0.00791 (19)	−0.00091 (16)
O1	0.0150 (7)	0.0182 (7)	0.0209 (7)	−0.0005 (6)	0.0107 (6)	−0.0016 (6)
O2	0.0156 (7)	0.0192 (7)	0.0201 (7)	0.0002 (6)	0.0096 (6)	−0.0001 (6)
O3	0.0135 (6)	0.0186 (7)	0.0189 (7)	−0.0002 (5)	0.0085 (6)	−0.0026 (5)
O4	0.0185 (7)	0.0215 (7)	0.0232 (7)	−0.0017 (5)	0.0143 (6)	−0.0020 (6)
O5	0.0187 (7)	0.0231 (7)	0.0160 (7)	0.0020 (6)	0.0091 (6)	0.0005 (6)
O6	0.0208 (7)	0.0179 (7)	0.0244 (7)	0.0034 (6)	0.0120 (6)	−0.0023 (6)
N11	0.0137 (7)	0.0195 (8)	0.0167 (8)	0.0010 (6)	0.0089 (7)	0.0004 (6)
N12	0.0166 (8)	0.0181 (8)	0.0206 (8)	−0.0013 (6)	0.0114 (7)	−0.0012 (6)
N13	0.0173 (8)	0.0185 (8)	0.0205 (8)	−0.0001 (6)	0.0126 (7)	−0.0002 (6)
N31	0.0182 (8)	0.0211 (8)	0.0199 (8)	0.0007 (6)	0.0109 (7)	0.0025 (7)
N32	0.0258 (9)	0.0194 (9)	0.0452 (12)	0.0010 (7)	0.0236 (9)	−0.0013 (8)
N33	0.0173 (8)	0.0216 (8)	0.0237 (9)	−0.0012 (7)	0.0136 (7)	−0.0013 (7)
C21	0.0145 (9)	0.0201 (9)	0.0180 (9)	−0.0002 (7)	0.0093 (8)	0.0003 (7)
C22	0.0187 (9)	0.0184 (9)	0.0306 (11)	−0.0013 (8)	0.0142 (9)	−0.0021 (8)
C23	0.0178 (9)	0.0227 (10)	0.0206 (9)	−0.0014 (8)	0.0126 (8)	−0.0018 (8)
C41	0.0172 (9)	0.0252 (10)	0.0172 (9)	−0.0006 (8)	0.0084 (8)	−0.0021 (8)
C42	0.0281 (11)	0.0196 (10)	0.0389 (13)	−0.0056 (8)	0.0224 (10)	−0.0052 (9)
C43	0.0219 (10)	0.0301 (11)	0.0227 (10)	−0.0027 (8)	0.0155 (9)	−0.0035 (8)
C51	0.0202 (10)	0.0200 (10)	0.0196 (10)	−0.0003 (7)	0.0109 (8)	−0.0025 (8)
C52	0.0184 (9)	0.0194 (9)	0.0264 (10)	−0.0033 (8)	0.0140 (9)	−0.0017 (8)
C53	0.0182 (9)	0.0237 (10)	0.0203 (9)	−0.0014 (8)	0.0119 (8)	−0.0036 (8)
C61	0.0302 (12)	0.0242 (11)	0.0319 (12)	0.0048 (9)	0.0169 (10)	0.0112 (9)
C62	0.0368 (14)	0.0281 (13)	0.078 (2)	0.0115 (11)	0.0379 (15)	0.0054 (13)
C63	0.0149 (9)	0.0286 (11)	0.0298 (11)	−0.0039 (8)	0.0127 (9)	−0.0026 (9)
C71	0.0353 (14)	0.0302 (13)	0.0618 (18)	0.0143 (11)	0.0252 (14)	0.0112 (12)
C72A	0.025	0.027 (2)	0.048 (3)	0.0004 (15)	0.0218 (14)	−0.0071 (18)
C72B	0.025	0.075 (6)	0.063 (5)	0.022 (3)	0.017 (3)	0.005 (4)
C73	0.0295 (11)	0.0316 (12)	0.0298 (11)	−0.0081 (9)	0.0211 (10)	−0.0042 (9)

### Geometric parameters (Å, º)

**Table:**

Ni1—N11	2.0630 (16)	C41—H41	0.9500
Ni1—N13	2.0667 (16)	C42—C52	1.354 (3)
Ni1—N12	2.0817 (17)	C42—H42	0.9500
Ni1—O2	2.1195 (15)	C43—C53	1.359 (3)
Ni1—O1	2.1485 (15)	C43—H43	0.9500
Ni1—O3	2.1502 (14)	C51—H51	0.9500
S1—O6	1.4574 (14)	C52—H52	0.9500
S1—O4	1.4878 (14)	C53—H53	0.9500
S1—O3	1.4902 (14)	C61—C71	1.492 (3)
S1—O5	1.4920 (14)	C61—H61A	0.9900
O1—H1A	0.84 (3)	C61—H61B	0.9900
O1—H1B	0.77 (3)	C62—C72A	1.499 (3)
O2—H2A	0.81 (3)	C62—C72B	1.512 (3)
O2—H2B	0.85 (3)	C62—H62A	0.9900
N11—C21	1.322 (3)	C62—H62B	0.9900
N11—C51	1.378 (3)	C62—H62C	0.9900
N12—C22	1.320 (3)	C62—H62D	0.9900
N12—C52	1.385 (3)	C63—C73	1.509 (3)
N13—C23	1.326 (3)	C63—H63A	0.9900
N13—C53	1.373 (3)	C63—H63B	0.9900
N31—C21	1.348 (3)	C71—H71A	0.9800
N31—C41	1.372 (3)	C71—H71B	0.9800
N31—C61	1.466 (3)	C71—H71C	0.9800
N32—C22	1.347 (3)	C72A—H72A	0.9800
N32—C42	1.378 (3)	C72A—H72B	0.9800
N32—C62	1.455 (3)	C72A—H72C	0.9800
N33—C23	1.345 (3)	C72B—H72D	0.9800
N33—C43	1.372 (3)	C72B—H72E	0.9800
N33—C63	1.471 (3)	C72B—H72F	0.9800
C21—H21	0.9500	C73—H73A	0.9800
C22—H22	0.9500	C73—H73B	0.9800
C23—H23	0.9500	C73—H73C	0.9800
C41—C51	1.360 (3)		
			
N11—Ni1—N13	91.81 (6)	C53—C43—N33	105.81 (18)
N11—Ni1—N12	97.97 (7)	C53—C43—H43	127.1
N13—Ni1—N12	94.83 (7)	N33—C43—H43	127.1
N11—Ni1—O2	172.09 (6)	C41—C51—N11	109.81 (18)
N13—Ni1—O2	89.27 (6)	C41—C51—H51	125.1
N12—Ni1—O2	89.74 (6)	N11—C51—H51	125.1
N11—Ni1—O1	88.13 (6)	C42—C52—N12	109.66 (18)
N13—Ni1—O1	176.45 (6)	C42—C52—H52	125.2
N12—Ni1—O1	88.70 (6)	N12—C52—H52	125.2
O2—Ni1—O1	90.31 (6)	C43—C53—N13	110.15 (18)
N11—Ni1—O3	88.30 (6)	C43—C53—H53	124.9
N13—Ni1—O3	89.68 (6)	N13—C53—H53	124.9
N12—Ni1—O3	172.14 (6)	N31—C61—C71	112.3 (2)
O2—Ni1—O3	83.87 (6)	N31—C61—H61A	109.1
O1—Ni1—O3	86.76 (6)	C71—C61—H61A	109.1
O6—S1—O4	110.10 (9)	N31—C61—H61B	109.1
O6—S1—O3	110.12 (8)	C71—C61—H61B	109.1
O4—S1—O3	108.54 (8)	H61A—C61—H61B	107.9
O6—S1—O5	111.04 (9)	N32—C62—C72A	113.7 (3)
O4—S1—O5	108.45 (8)	N32—C62—C72B	115.8 (3)
O3—S1—O5	108.52 (8)	N32—C62—H62A	108.8
Ni1—O1—H1A	101 (2)	C72A—C62—H62A	108.8
Ni1—O1—H1B	122 (2)	N32—C62—H62B	108.8
H1A—O1—H1B	101 (3)	C72A—C62—H62B	108.8
Ni1—O2—H2A	106 (2)	H62A—C62—H62B	107.7
Ni1—O2—H2B	116 (2)	N32—C62—H62C	108.3
H2A—O2—H2B	104 (3)	C72B—C62—H62C	108.3
S1—O3—Ni1	130.19 (8)	N32—C62—H62D	108.3
C21—N11—C51	105.50 (17)	C72B—C62—H62D	108.3
C21—N11—Ni1	121.44 (14)	H62C—C62—H62D	107.4
C51—N11—Ni1	133.00 (14)	N33—C63—C73	111.69 (18)
C22—N12—C52	105.42 (17)	N33—C63—H63A	109.3
C22—N12—Ni1	123.31 (14)	C73—C63—H63A	109.3
C52—N12—Ni1	130.90 (13)	N33—C63—H63B	109.3
C23—N13—C53	105.29 (16)	C73—C63—H63B	109.3
C23—N13—Ni1	128.18 (14)	H63A—C63—H63B	107.9
C53—N13—Ni1	126.53 (13)	C61—C71—H71A	109.5
C21—N31—C41	107.37 (17)	C61—C71—H71B	109.5
C21—N31—C61	125.43 (18)	H71A—C71—H71B	109.5
C41—N31—C61	127.20 (18)	C61—C71—H71C	109.5
C22—N32—C42	107.12 (18)	H71A—C71—H71C	109.5
C22—N32—C62	123.8 (2)	H71B—C71—H71C	109.5
C42—N32—C62	128.9 (2)	C62—C72A—H72A	109.5
C23—N33—C43	107.43 (17)	C62—C72A—H72B	109.5
C23—N33—C63	125.87 (18)	H72A—C72A—H72B	109.5
C43—N33—C63	126.65 (18)	C62—C72A—H72C	109.5
N11—C21—N31	111.34 (17)	H72A—C72A—H72C	109.5
N11—C21—H21	124.3	H72B—C72A—H72C	109.5
N31—C21—H21	124.3	C62—C72B—H72D	109.5
N12—C22—N32	111.58 (18)	C62—C72B—H72E	109.5
N12—C22—H22	124.2	H72D—C72B—H72E	109.5
N32—C22—H22	124.2	C62—C72B—H72F	109.5
N13—C23—N33	111.33 (18)	H72D—C72B—H72F	109.5
N13—C23—H23	124.3	H72E—C72B—H72F	109.5
N33—C23—H23	124.3	C63—C73—H73A	109.5
C51—C41—N31	105.98 (18)	C63—C73—H73B	109.5
C51—C41—H41	127.0	H73A—C73—H73B	109.5
N31—C41—H41	127.0	C63—C73—H73C	109.5
C52—C42—N32	106.21 (19)	H73A—C73—H73C	109.5
C52—C42—H42	126.9	H73B—C73—H73C	109.5
N32—C42—H42	126.9		
			
O6—S1—O3—Ni1	−172.84 (10)	C23—N33—C43—C53	−0.2 (2)
O4—S1—O3—Ni1	−52.25 (13)	C63—N33—C43—C53	−177.64 (19)
O5—S1—O3—Ni1	65.42 (13)	N31—C41—C51—N11	0.4 (2)
C51—N11—C21—N31	0.3 (2)	C21—N11—C51—C41	−0.5 (2)
Ni1—N11—C21—N31	−177.08 (12)	Ni1—N11—C51—C41	176.55 (14)
C41—N31—C21—N11	−0.1 (2)	N32—C42—C52—N12	0.3 (3)
C61—N31—C21—N11	179.85 (19)	C22—N12—C52—C42	−0.4 (2)
C52—N12—C22—N32	0.3 (3)	Ni1—N12—C52—C42	172.68 (15)
Ni1—N12—C22—N32	−173.42 (15)	N33—C43—C53—N13	0.4 (2)
C42—N32—C22—N12	−0.1 (3)	C23—N13—C53—C43	−0.4 (2)
C62—N32—C22—N12	176.4 (2)	Ni1—N13—C53—C43	−179.64 (14)
C53—N13—C23—N33	0.3 (2)	C21—N31—C61—C71	114.2 (3)
Ni1—N13—C23—N33	179.50 (13)	C41—N31—C61—C71	−65.9 (3)
C43—N33—C23—N13	−0.1 (2)	C22—N32—C62—C72A	130.5 (3)
C63—N33—C23—N13	177.42 (18)	C42—N32—C62—C72A	−53.8 (4)
C21—N31—C41—C51	−0.2 (2)	C22—N32—C62—C72B	−110.8 (5)
C61—N31—C41—C51	179.9 (2)	C42—N32—C62—C72B	64.9 (5)
C22—N32—C42—C52	−0.1 (3)	C23—N33—C63—C73	−111.6 (2)
C62—N32—C42—C52	−176.4 (2)	C43—N33—C63—C73	65.4 (3)

### Hydrogen-bond geometry (Å, º)

Cg1 is the centroid of the N13–C23–N33–C43–C53 ring and Cg2 is the centroid of the N12–C22–N32–C42–C52 ring

**Table:**

*D*—H···*A*	*D*—H	H···*A*	*D*···*A*	*D*—H···*A*
O1—H1*A*···O4	0.84 (3)	1.88 (3)	2.706 (2)	170 (3)
O1—H1*B*···O5^i^	0.77 (3)	2.02 (3)	2.786 (2)	173 (3)
O2—H2*B*···O4^i^	0.85 (3)	1.88 (3)	2.720 (2)	171 (3)
O2—H2*A*···O5	0.81 (3)	2.00 (3)	2.791 (2)	165 (3)
C22—H22···O5^i^	0.95	2.60	3.511 (3)	162
C23—H23···O6^ii^	0.95	2.56	3.409 (3)	150
C52—H52···O6^ii^	0.95	2.42	3.315 (3)	157
C73—H73*B*···O6^iii^	0.98	2.40	3.347 (3)	163
C61—H61*A*···*Cg*1^iv^	0.99	2.80	3.779 (3)	169
C61—H61*B*···*Cg*2^v^	0.99	2.97	3.816 (3)	144

Symmetry codes: (i) −*x*, −*y*, −*z*; (ii) −*x*+1, *y*+1/2, −*z*+1/2; (iii) −*x*+1, −*y*, −*z*; (iv) −*x*+1, *y*−1/2, −*z*+1/2; (v) −*x*+1, −*y*, −*z*+1.
